# Circulating tumor cell heterogeneity in neuroendocrine prostate cancer by single cell copy number analysis

**DOI:** 10.1038/s41698-021-00211-1

**Published:** 2021-08-12

**Authors:** Vincenza Conteduca, Sheng-Yu Ku, Luisa Fernandez, Angel Dago-Rodriquez, Jerry Lee, Adam Jendrisak, Megan Slade, Cole Gilbertson, Jyothi Manohar, Michael Sigouros, Yipeng Wang, Ryan Dittamore, Rick Wenstrup, Juan Miguel Mosquera, Joseph D. Schonhoft, Himisha Beltran

**Affiliations:** 1 Dana Farber Cancer Institute and Harvard Medical School, Boston, MA USA; 2 IRCCS Istituto Romagnolo per lo Studio dei Tumori (IRST) “Dino Amadori”, Meldola, Italy; 3 Epic Sciences, Inc., San Diego, CA USA; 4 Weill Cornell Medicine, New York, NY USA

**Keywords:** Tumour heterogeneity, Prostate cancer

## Abstract

Neuroendocrine prostate cancer is an aggressive variant of prostate cancer that may arise *de novo* or develop from pre-existing prostate adenocarcinoma as a mechanism of treatment resistance. The combined loss of tumor suppressors *RB1, TP53,* and *PTEN* are frequent in NEPC but also present in a subset of prostate adenocarcinomas. Most clinical and preclinical studies support a trans-differentiation process, whereby NEPC arises clonally from a prostate adenocarcinoma precursor during the course of treatment resistance. Here we highlight a case of NEPC with significant intra-patient heterogeneity observed across metastases. We further demonstrate how single-cell genomic analysis of circulating tumor cells combined with a phenotypic evaluation of cellular diversity can be considered as a window into tumor heterogeneity in patients with advanced prostate cancer.

## Introduction

Cell-to-cell heterogeneity is a major driver of cancer evolution, progression, and drug resistance. Neuroendocrine prostate cancer (NEPC) is an aggressive subtype of prostate cancer with variant histology, characterized by poorly differentiated neuroendocrine carcinoma morphology (ie., small cell or large cell carcinoma) and high proliferation indices^1^. NEPC may arise *de novo* or develop from prostate adenocarcinoma as a mechanism of treatment resistance^2,3^. During this progression, mixed histologic features may be observed with both high-grade prostate adenocarcinoma and neuroendocrine carcinoma present. In NEPC, expression of the androgen receptor (AR) and prostate-specific antigen (PSA) are often negative; however, in some cases, particularly in mixed cases or hybrid tumors with overlapping features, AR can be expressed with or without PSA^1,2,4,5^. Classical neuroendocrine markers such as chromogranin, synaptophysin, CD56 are also expressed in NEPC, though not universally. The combined loss of tumor suppressors *RB1, TP53, PTEN* are frequent in NEPC and are also present in a subset of prostate adenocarcinoma^5^. In treatment-related NEPC, most clinical and preclinical studies support a trans-differentiation process where NEPC arises clonally from a prostate adenocarcinoma precursor^3,6^. This trans-differentiation occurs through a process of lineage plasticity, whereby tumor cells lose their luminal prostate identity including AR signaling dependence, and can acquire a neuroendocrine lineage program. Our recent circulating tumor DNA (ctDNA) genomic analyses suggest that at some point during disease progression, there may also be clonal selection with the emergence of a dominant resistance clone in NEPC^7^. Here we highlight a patient case to illustrate the degree of intra-patient tumor heterogeneity that can be seen in NEPC. We then use single-cell genomic analysis of circulating tumor cells (CTCs) in this patient and additional patients to further explore the contribution of cellular diversity in NEPC.

## Results

### Case report

**Fig. 1:**
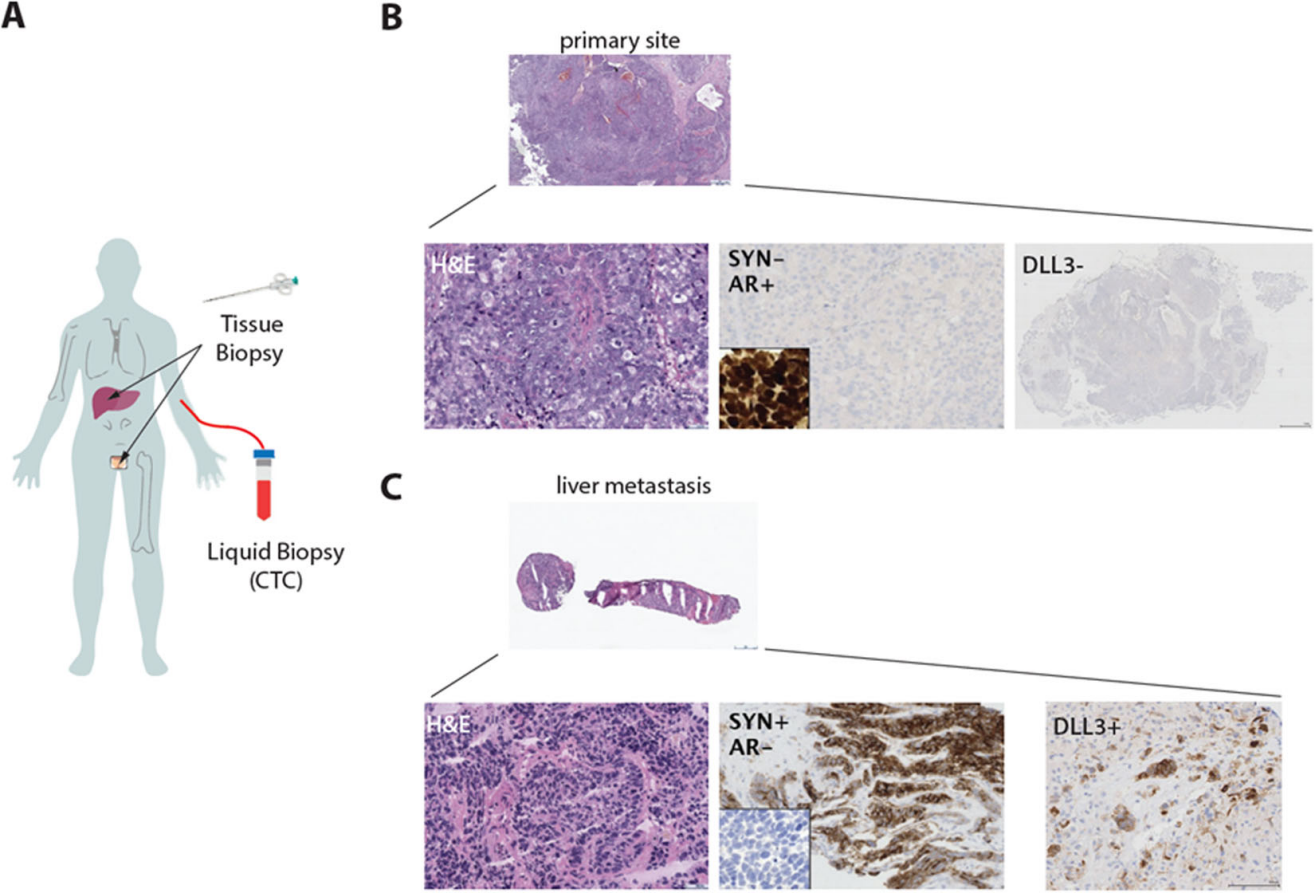
Intra-tumoral heterogeneity in a patient with newly diagnosed metastatic prostate cancer. **A** Schematic illustrating sites of metastasis for NEPC case report and the analysis of tissue and liquid biopsy. **B** Primary prostate adenocarcinoma with immunohistochemical (IHC) staining positive for androgen receptor (AR) and negative for the neuroendocrine markers synaptophysin (SYP). The primary tumor also lacks expression of Delta-like protein 3 (DLL3) by IHC. **C** Metastatic liver biopsy showing neuroendocrine prostate cancer (NEPC) morphology, AR-negative and SYP positive by IHC. DLL3 is expressed in the NEPC tumor cells. Scale bars, 100 µm.

An 82-year-old man presented with extensive lytic bone and liver metastases and an enlarged prostate gland with the extension of a prostate mass into the bladder (Fig. 1A). His serum PSA was 6.23 ng/mL. He underwent a transurethral resection, which revealed high-grade prostate adenocarcinoma (Fig. 1B). He also underwent a liver biopsy at the same time-point, which demonstrated metastatic high-grade adenocarcinoma with extensive neuroendocrine differentiation (Fig. 1C). Immunohistochemical stains of the primary prostate tumor demonstrated positive staining of the AR, NKX3.1, and PSMA, and negative staining for neuroendocrine (NE) markers synaptophysin, chromogranin A, and DLL3. His liver biopsy was negative for AR, NKX3.1, and p501S expression by immunohistochemistry and expressed the neuroendocrine markers synaptophysin and DLL3. Based on his aggressive clinical presentation and pathologic features of both adenocarcinoma and NEPC, the patient was treated with androgen deprivation therapy (ADT), carboplatin and docetaxel chemotherapy. Imaging after cycles 4 and 6 of chemotherapy showed a radiographic response and he had an overall improvement in symptoms.

Whole-exome and targeted sequencing of his pre-treatment primary and metastatic biopsies were performed. Both his primary tumor and his liver biopsy shared genomic alterations including loss of *RB1* (c.1027_1028delCT, p.L343fs*3 and area of broad deletion involving this gene) and *PTEN* (homozygous deletion) and *TP53* mutation (c.817C > T, p.R273C). Both tumors (primary and metastatic) were also assessed for expression of the neuroendocrine marker delta-like ligand 3 (DLL3) by immunohistochemistry (clone SP346, Ventana-Roche Diagnostics, Indianapolis, IN, USA). DLL3 is a cell surface marker found to be expressed in 76.6% of NEPC and 12.5% of castration-resistant adenocarcinomas, but not typically present in hormone naïve localized prostate adenocarcinoma or benign tissues^8^. DLL3 is also expressed in small cell lung cancer and other neuroendocrine carcinomas^9^. For this patient, 97% of total tumor cells in his liver biopsy expressed DLL3 at 3+ intensity (Fig. 1B). None of the tumor cells in his primary tumor were positive for DLL3 (Fig. 1A).

**Fig. 2:**
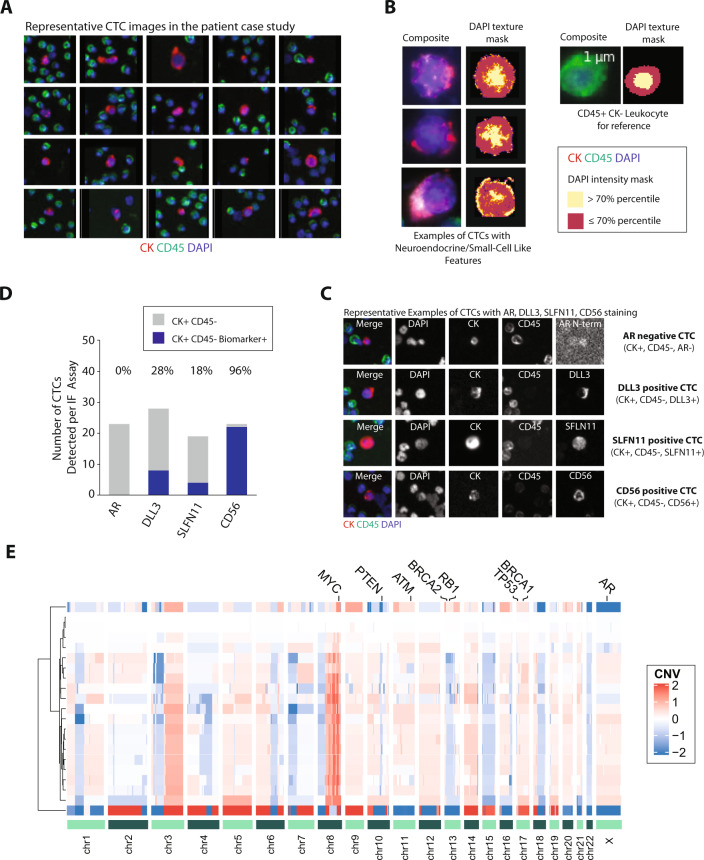
Landscape of CTCs in our patient with metastatic prostate cancer with both adenocarcinoma and NEPC morphologies. **A** Representative images of CK+, CD45− CTCs detected in our patient. **B** Representative CTCs with small-cell-like features (N/C ratio greater than 0.8 and area less than approximately 2× that of white blood cells. A mask of the DAPI channel, showing the top 30% in terms of intensity is shown to better visualize the observed texture patterns. A leukocyte is shown for comparison. **C** Representative images of CTCs stained with a 4th marker for AR, DLL3, SLFN11, or CD56. In the three-color composite image, blue represents 4′,6-diamidino-2-phenylindole (DAPI), and red is used for CK, green for CD45, and white for DLL3. Other images show the gray-scale intensity for each channel. **D** The number of total CTCs and CTCs expressing either AR, DLL3, SLFN11, or CD56. **E** Analysis of copy number profiles by single-cell low-pass whole-genome sequencing of CTCs.

Whole blood (10 ml) was also collected pre-treatment using Streck tubes and shipped to Epic Sciences for processing using protocols previously described^10^. CTCs slides were stained for DNA (DAPI), whole blood cell linage marker (CD45), and cytokeratins (CK) to determine epithelial lineage (Supplementary Table 1), as well as AR, SLFN11, and the neuroendocrine markers CD56 and DLL3 in separate assays (four-channels in total for each assay) (Supplementary Table 1). Prior work in assay development revealed CD56 to stain CTCs with less background than synaptophysin on the Epic Sciences platform^11^. SLFN11 is a marker that is associated with platinum sensitivity in prostate cancer^12^ and other cancers^13^. The atypical intra-patient heterogeneity of our case, with differences observed between primary untreated prostate and liver biopsies at the same timepoint, was also captured by CTCs (Fig. 1). Ninety-nine CTCs, defined as the presence of CK+, CD45− cells were detected in total in the pre-treatment blood sample and were found to be morphologically heterogeneous (representative images in Fig. 2A). Qualitatively, several CTCs had characteristic features of small-cell NEPC-like morphology that included small-size, circular shape, high nuclear-to-cytoplasm ratio, and salt-and-pepper-like or textured chromatin observed by DAPI stain, akin to criteria used in a small-cell carcinoma diagnosis in tissues. These CTC criteria for NEPC were previously described in Beltran et al^10^ and recently applied to a prospective cohort in Brown et al^14^. A DAPI texture mask is shown to illustrate the observed textured chromatin compared with a leukocyte for reference. Pixels are colored according to an intensity cutoff to approximate areas of euchromatin (≤70th percentile) and heterochromatin (>70th percentile) (Fig. 2B).

In addition to the standard assessment of epithelial lineage and morphology, AR protein (full length and splice-variants, stained with an N-terminus specific antibody) was detected in 0% of CTCs, DLL3 was detected in 27.6%, SLFN11 in 22.2%, and CD56 in 96% of CTCs in our patient (Fig. 2C, D). SLFN11 mRNA was confirmed to be expressed in his liver metastasis by RNA sequencing and not expressed in his primary prostate tumor. Single-cell low-pass whole-genome sequencing of 21 of his CTCs was performed as previously described^15^. Of the CTCs sequenced, *RB1* loss was detected in 4 (19%), *TP53* loss in 17 (81%), and *PTEN* loss in 17 (81%) (Fig. 2E). All CTCs with *RB1* loss had concurrent *TP53* and *PTEN* loss. Overall, we observed a strong correlation in the state of heterogeneity (ie higher similarity) between CTCs and his liver biopsy compared with the primary tumor biopsy.

The patient responded initially to platinum chemotherapy. He subsequently progressed after five months with new pleural metastases and progression of lytic bone, lung, and liver metastases. He died approximately six months from the time of his initial diagnosis. The autopsy revealed widely metastatic prostatic adenocarcinoma with neuroendocrine, sarcomatoid, giant cell, and squamous differentiation. Metastatic sites included lungs, pleura, liver, bone, penis, bladder, adrenal gland, brain, and para-aortic and subcarinal lymph nodes.

This uncommon and aggressive presentation of prostate cancer with diverse histologic features observed across metastases at diagnosis and at the time of autopsy highlights the contribution of intra-patient heterogeneity in influencing patient management and supports the ability to detect this heterogeneity using CTCs. Of note, most newly diagnosed patients with prostate cancer do not have two synchronous tumor biopsies at initial presentation. If his treatment was based only on his prostate biopsy, he may have been treated very differently for metastatic prostate adenocarcinoma (ie. androgen deprivation therapy with either docetaxel or potent hormonal therapy) and may not have received upfront platinum chemotherapy. CTCs were, therefore, able to capture tumor heterogeneity that may have otherwise been missed on single-site biopsy.

### CTC genomic landscape by single-cell copy number analysis in additional patients

To further expand upon the potential utility of CTCs, and particularly genomic heterogeneity that may be seen or conserved across metastases, we evaluated CTC morphology and genotype in an additional 11 patients with advanced prostate cancer along with a matched metastatic tumor biopsy confirming pathologic features [4 biopsy-confirmed castration-resistant adenocarcinoma (CRPC-Adeno) and 7 biopsy-confirmed NEPC] (Supplementary Data). NEPC cases included two patients with a pure small cell prostate carcinoma and five with mixed histology (both adenocarcinoma and small-cell neuroendocrine carcinoma). Two NEPC patients were further classified as *de novo*, meaning at the time of NEPC diagnosis they had no prior diagnosis or treatment for prostate adenocarcinoma. Most (5/7) NEPC patients were treated with platinum-based chemotherapy using typical regimens for SCLC. The median number of therapeutic lines before CTC collection was two (range 1–5). Pre-treatment median PSA in NEPC and CRPC-Adeno patients was 0.18 and 247 ng/ml, respectively. Clinical features are summarized in Table 1.

**Table 1:** Clinical and molecular features of patients.

Patient id	Age (years)	Histology	Radical prostatectomy and/or radiotherapy	Primary ADT	Number of therapies before CTC collection	Type of tp at time of CTC collection	Serum CGA and /or NSE	Serum PSA (ng/ml)	Presence of visceral metastasis	Number of TSG (no = 0, single = 1, double = 2, triple = 3)
10	79	Adeno-NE	No	Yes	2	Cisplatin etoposide	High	0.18	Yes	1
11	62	CRPC-Adeno	Yes	Yes	3	Abiraterone	Normal	165	No	0
12	69	Small cell	No	Yes	1	Cisplatin	High	0.01	Yes	2
26	68	Small cell	Yes	Yes	3	Aurora A kinase inhibitor	High	0.008	Yes	2
31	74	Adeno-NE	No	Yes	2	Carboplatin etoposide	High	1.39	Yes	3
33	68	CRPC-Adeno	Yes	Yes	4	Docetaxel	Normal	329	No	1
35	59	Adeno-NE	Yes	No	1	Cisplatin etoposide	High	0.98	No	2
36	70	Adeno-NE	Yes	Yes	5	Rovalpituzumab tesirine	High	0.06	Yes	1
37	58	CRPC-Adeno	No	Yes	2	Enzalutamide	Normal	27.2	No	0
38	79	Adeno-NE	Yes	Yes	1	Cisplatin etoposide	High	0.02	Yes	2
41	65	CRPC-Adeno	Yes	Yes	4	Enzalutamide	Normal	7.85	No	1

Abbreviations. *Adeno-NE* adenocarcinoma with neuroendocrine differentiation, *ADT* androgen deprivation therapy, *CGA* chromogranin A, *CRPC-Adeno* castration-resistant prostate adenocarcinoma, *CTC* circulating tumor cell, *NSE* neuron-specific enolase, *TSG* tumor suppressor genes.

**Fig. 3:**
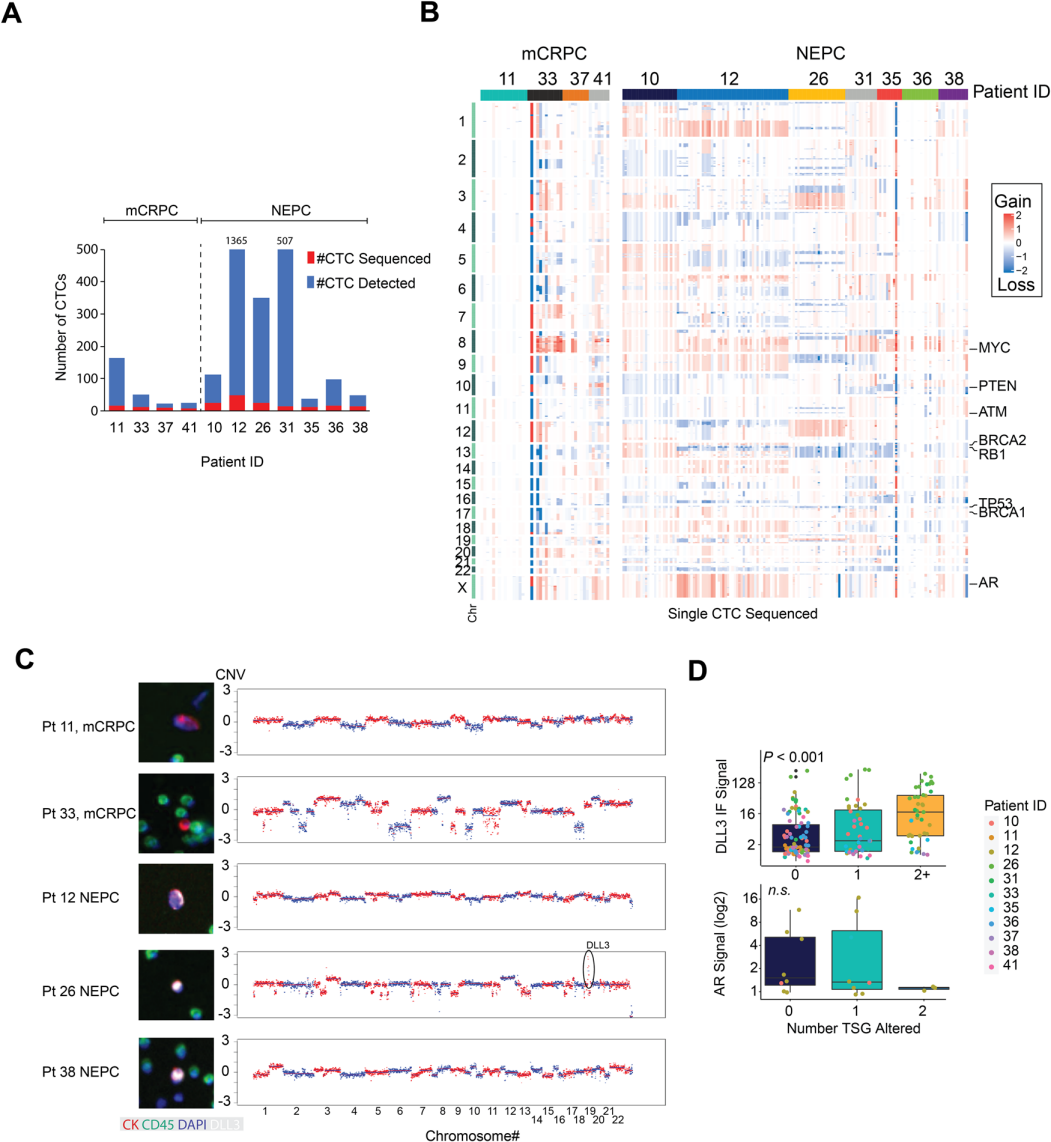
Genomics of single-cell CTCs. **A** The number of CTCs detected vs single-cell sequenced. **B** Copy number profiles across chromosomes for each CTC sequenced. **C** Intensity of DLL3 or AR protein expression assess my immunofluorescence compared tumor suppressor loss (PTEN, RB1, and P53) in single CTCs. **D** Representative copy-number profiles and corresponding CTC image from 5/11 patients.

Between the two groups, more CTCs were detected in the NEPC patient blood samples compared with CRPC-Adeno patients (median CTC/mL 33 vs 16 respectively; Fig. 3A). Further, CD56 was assessable in three NEPC patients (pts 10, 12, and 26) in which all had expression, and one CRPC-Adeno (pt 11) that did not have CD56 expression. Qualitatively, CTC morphologic features from NEPC were similar to previously described^10,14^. Of the detectable CTCs, 191 in total were isolated from the slide, and the genomic material was subjected to single-cell whole genome amplification and low-pass sequencing (Fig. 3A). We evaluated for copy-number alterations for regions involving commonly altered prostate cancer genes, including the *AR* gene, tumor suppressors genes (*RB1, TP53,* and *PTEN*), and homologous recombination (HR) repair genes (*BRCA1, BRCA2,* and *ATM*). We analyzed a median number of CTCs per patient of 14 (range 5−49). The percentages of single cells with specific copy number alterations in relevant gene regions are shown in Table 2. In addition, CNAs observed in single CTCs were compared with whole-exome sequencing (WES) data of patient-matched metastatic biopsies (Table 2).

**Table 2:** Gene region alterations in single CTCs and comparison to tissue biopsy whole-exome sequencing (WES).

*CTC sequencing*											
Number of CTCs detected and single-cell sequenced											
Patient ID	mCRPC				NEPC						
	Pt 11	Pt 33	Pt 37	Pt 41	Pt 10	Pt 12	Pt 26	Pt 31	Pt 35	Pt 36	Pt 38
Total CTCs	164	12	22	25	113	1365	351	57	38	98	48
CTCs sequenced	16	5	9	7	24	49	25	14	11	16	13
Gene regions evaluated, % of CTCs with gene region alteration											
RB1 loss						47	64	14			
TP53 loss		33			42	55	8	5	45	31	24
PTEN loss				29				43	45		24
AR gain		83									
NCOA2 gain		75	33	43		37		71	27	19	
MYC gain	13	83	67	71	38	65	12	79	73	44	38
AURKA gain		42		29		12	8	36			24
UBE2C gain		42				14	8	36			24
DLL3 gain							92	21			15
NCOR1 loss		33			29	6	56	79	18		24
ATM loss							52	14		13	
BRCA1 loss		25									
BRCA2 loss		25		57			8	5	55	31	4
CDKN1B loss					25	65					
CDKN2A loss	13	17		71		6	88	36			
ERF loss		17		71		37	8				
MAP2K4 loss		33	33		67	12	8	64		19	38
CDH1 loss		5			54			36	82	25	62
TP53/RB1 loss						37	6	14			
TP53/PTEN loss								43	27		
TP53/PTEN/RB1 loss								14			
NCOA2/MYC gain		75	33	43		29		5	27	19	
AR/NCOA2 gain		75									
*Tissue whole exome sequencing (WES)*											
Tissue WES available	No	Yes	Yes	Yes	Yes	Yes	Yes	Yes	No	Yes	No
AR	na	AMP							na		na
RB1 loss	na		Deletion			Deletion	Deletion	Deletion	na	Deletion	na
TP53 loss	na		Deletion		Deletion		Deletion	Deletion	na	Deletion	na
PTEN loss	na			Deletion					na	Deletion	na
MYC gain	na		AMP						na	AMP	na
Any BRCA1/BRCA2/ATM	na					Deletion	Deletion	Deletion	na	Deletion	na

Abbreviations. *AMP* amplification, *CTC* circulating tumor cell, *mCRPC* metastatic castration-resistant prostate adenocarcinoma, *na* not available, *NEPC* neuroendocrine prostate cancer, *Pt* patient.

Copy-number profiles are shown in Fig. 3B; marked inter- and intra-patient heterogeneity was observed. For instance, CTCs from both metastatic CRPC-Adeno and NEPC were observed to have common alterations such as gain in the region of chromosome 8 containing the MYC gene, while the NEPC patient 26 was observed to have a copy-number pattern entirely unique from the other patients. Further within each patient, CTC profiles were generally clonal, however, certain gene region alterations were only observed in a fraction of CTCs; e.g., *RB1* loss in 14% of sequenced CTCs in patient 31 versus 64% in patient 26, suggesting that the latter may be part of a branch in the evolution from earlier stages (Table 2). Representative profiles and corresponding CTC images are shown in Fig. 3C.

Overall, the combined loss of tumor suppressor genes *RB1, TP53,* and/or *PTEN* in single CTCs was associated with higher expression of the NE marker DLL3 (Kruskal-Wallis *P* < 0.001, respectively) and lower expression of AR (Fig. 3D). The combined loss of tumor suppressor gene regions occurred a greater number of CTCs in NEPC patients, while conversely AR amplification in single CTCs was identified in one CRPC-adeno patient and was not observed in any CTCs detected in NEPC (Table 2). Additional observations at the single CTC level were that loss of *RB1* and *CDH1* were near mutually exclusive, and gain of *NCOA2* and *MYC* were mostly correlated. Loss of the nuclear receptor corepressor *NCOR1* and tumor suppressor *MAP2K4* were commonly observed in NEPC. Other CNAs (e.g*., CDK4/6*, *AURKA*, *AKT2* gain, and *MYC* amplification) showed similar qualitative trends as seen with tumor suppressor loss, with a higher number of alterations across and within single CTCs in individuals with NEPC [Data segment files and gene-level copy number variation (CNV) data can be found in the Supplementary Information]. Gene region copy-number alterations were generally similar compared with tissue WES data (Table 2). As an example, *AR* amplification in CRPC patient 33 was also observed, and 3/5 patients with *RB1* loss in tissue also had *RB1* loss in single CTCs. Data for *AR*, tumor suppressors, and *BRCA1/2* and *ATM* are summarized in Table 2.

## Discussion

Recent genomic landscape studies have identified distinct molecular subclasses of advanced prostate cancer providing insights into inter-patient heterogeneity and emerging drivers of treatment response and resistance^2,5,17–20^. However, the role of intra-patient heterogeneity is still not fully established. Gundem et al.^21^ reported that in some cases, with up to ten separate metastatic prostate cancer sites sampled in the same patient, no two sites had the same clonal or subclonal composition, suggesting significant intrapatient heterogeneity occurs within this disease. Incorporating new technologies to capture molecular heterogeneity including those involving liquid biopsies may be important in translating the findings of such studies into the clinic. Furthermore, the diagnosis of NEPC currently relies on a tumor biopsy, which is invasive for patients and limited in resolving spatiotemporal heterogeneity, as shown in our case.

As highlighted in this case, CTCs detected tumor heterogeneity that would likely not have been appreciated in the clinic as most patients do not get two biopsies at the time of their initial diagnosis. If his treatment was based on his first adenocarcinoma biopsy, he may not have received upfront platinum chemotherapy. Platinum chemotherapy in prostate cancer is typically considered for patients with small cell NEPC^22^, aggressive variant clinical features^23^, or those with *BRCA* alterations^24^ .

Liquid biopsies offer great potential for the management of patients with prostate cancer. Despite the numerous techniques and experimental approaches that have been established in the field, their common objective is to develop a useful, sensitive, specific, and real-time monitoring system using minimally invasive samples, which can be easily transferred into clinical practice. It is probable that ctDNA analysis should be chosen for analysis of mutations, copy number aberrations, and DNA methylation changes, whereas CTC analysis provides the unique opportunity to study whole cells, thus allowing for additional mRNA and protein-based molecular profiling. Therefore, CTCs and ctDNA may have complementary roles as cancer biomarkers and might be used in parallel in the setting of castration-resistant prostate cancer, particularly for NEPC detection, prediction of treatment responses, or detection of disease progression. In our patient case with mixed morphologic features across tumor sites, for instance, the dominant cell type detected through CTC analysis was concordant with the neuroendocrine tumor biopsy.

Single-cell CTC genomics has the capacity to monitor tumor clone dynamics and disease evolution, along with correlation of genomics with phenotypic features of aggressive variants and assessment of other biomarkers potentially representative of multiple metastases. In agreement with recent work in aggressive variant prostate cancer^15^ we found that combined loss of tumor suppressor genes is more frequent in NEPC compared with CRPC-Adeno. Here we discovered that this often co-occurs in individual tumor cells. The detection of NE markers in CTCs and of these clonal CNAs in tumor suppressor genes at the single-cell level may lead to a comprehensive characterization of tumor aggressiveness, considering that the presence of alterations in more than two tumor suppressor genes in single CTCs was associated with poor prognosis.

The small number of included patients due to the requirement of matched metastatic biopsy tissue, heterogeneous therapies, and the lack of a serial monitoring of CTCs represent the most important limitations of this study that did not permit performing intra- or inter-survival analyses between different histologies and types of therapies. In addition, we only looked at CNAs in CTCs, and may have missed relevant AR, TP53, or other mutations. Lastly, we performed low-pass WGS, which is limited in its detection of all CNAs, especially if they are smaller than 100 kb. However, this study provided new insights into the ability of single-cell CTC sequencing to molecularly characterize heterogeneous populations within patients with advanced prostate cancer and to improve our understanding of neuroendocrine phenotype as a complex mechanism of therapeutic resistance. This work also further supports the feasibility of detecting potential predictive biomarkers such as DLL3^8,25^, which may have relevance for DLL3-targeted therapies (eg., NCT04471727, NCT04702737) and SLFN11 with potential implications for platinum-based chemotherapy or PARP inhibitors^12,26,27^. Future larger studies could help elucidate how molecular alterations emerge during the course of therapy and during the transition from adenocarcinoma to NEPC.

## Methods

### Cohort description and pathology classification

Patients provided written informed consent for the collection of tumor and blood samples. Biopsies and molecular testing were performed in accordance with Institutional Review Board-approved protocols (IRB#1305013903 and IRB#1502015936). NEPC was defined by the presence of either pure small cell carcinoma or mixed adenocarcinoma with neuroendocrine differentiation (adeno-NE)^28^. All other patients had a metastatic biopsy confirming prostate adenocarcinoma histology and clinical features of castration resistance defined according to Prostate Cancer Clinical Trials Working Group 3 (PCWG3) criteria (CRPC-Adeno)^29^. The study was conducted in accordance with the Declaration of Helsinki and the Good Clinical Practice guidelines. Clinical and demographic information was collected by medical record review. PCWG3 criteria^29^ was used to assess clinical, biochemical, and radiographic response to therapy.

### Immunohistochemistry (IHC)

IHC was performed on deparaffinized formalin-fixed paraffin-embedded sections using a Bond III automated immunostainer (Leica Microsystems). Bond Epitope Retrieval Solution 1 (ER1) (at pH 6) or ER2 was used with heat-mediated antigen retrieval. The following antibodies and conditions were used: SYP (SP11, Thermo Fisher Scientific; 1:100 dilution, ER2), AR (F39.4.1, BioGenex; 1:800 dilution with casein, ER1), DLL3 (SP347, Spring Bioscience).

### Biopsy sequencing

Metastatic tumor genomic status of select genes (ie., AR, TP53, RB1, BRCA2, BRCA1, and ATM) was collected from a review of WES data obtained through a clinical CLEB/CLIA compliant tumor/normal WES assay developed at WCM (EXaCT-1; IPM-Exome-pipeline, version 0.9)^30,31^. In addition, for cases with adequate fresh/frozen tissue, RNA sequencing data was evaluated to assess the expression of SLFN11^12^.

### Circulating tumor cell analysis

CTC detection, immunofluorescence analysis, and single-cell CNV analysis were performed using the Epic Sciences platform^16,32^. Post phlebotomy blood was sent overnight to Epic Sciences and nucleated cells were deposited onto glass pathology slides at a density of 3 million cells per slide. For each blood sample, 12−16 slides were made and then bio-banked at −80 °C. Upon analysis, slides were stained in a four-channel assay with antibodies for CD45, CK, and the small-molecule 4′,6-diamidino-2-phenylindole (DAPI) along with staining for one other marker that included either the AR (N-terminus specific antibody), SLFN11, DLL3, or CD56^8,12,33^. Each cell on the stained slide was then imaged and rare cells were detected using proprietary algorithms and confirmed by trained human technicians. CTCs were then isolated from the stained glass slides and genomic material was subject to whole-genome amplification and sequenced on an Illumina NextSeq500. The resultant data was then analyzed for CNV across known prostate cancer-specific gene alterations. The method and analysis pipeline have been published^16^.

### Reporting summary

Further information on research design is available in the Nature Research Reporting Summary linked to this article.

## Supplementary information

Supplementary Data 1

Supplementary Data 2

Supplementary Data 3

Supplementary Data 4

Supplementary Data 5

Supplementary Data 6

Supplementary Data 7

Supplementary Data 8

Supplementary Data 9

Supplementary Data 10

Supplementary Data 11

Supplementary Data 12

Supplementary Data 13

Supplementary Data 14

Reporting Summary

Supplementary Information

## Data Availability

The data generated for this study are described in the Supplementary files and are available via dbGaP under the accession number phs002462.v1.p1.
