# Author Correction: Circulating tumor cell heterogeneity in neuroendocrine prostate cancer by single cell copy number analysis

**DOI:** 10.1038/s41698-021-00227-7

**Published:** 2021-09-13

**Authors:** Vincenza Conteduca, Sheng-Yu Ku, Luisa Fernandez, Angel Dago-Rodriquez, Jerry Lee, Adam Jendrisak, Megan Slade, Cole Gilbertson, Jyothi Manohar, Michael Sigouros, Yipeng Wang, Ryan Dittamore, Rick Wenstrup, Juan Miguel Mosquera, Joseph D. Schonhoft, Himisha Beltran

**Affiliations:** 1 Dana Farber Cancer Institute and Harvard Medical School, Boston, MA USA; 2 IRCCS Istituto Romagnolo per lo Studio dei Tumori (IRST) “Dino Amadori”, Meldola, Italy; 3 Epic Sciences, Inc., San Diego, CA USA; 4 Weill Cornell Medicine, New York, NY USA

**Keywords:** Tumour heterogeneity, Prostate cancer

Correction to: *npj Precision Oncology* 10.1038/s41698-021-00211-1, published online 12 August 2021

The original version of this Article mistakenly omitted panel E in Fig. 2. This has now been corrected in the PDF and HTML versions of the Article.

**Figure:**
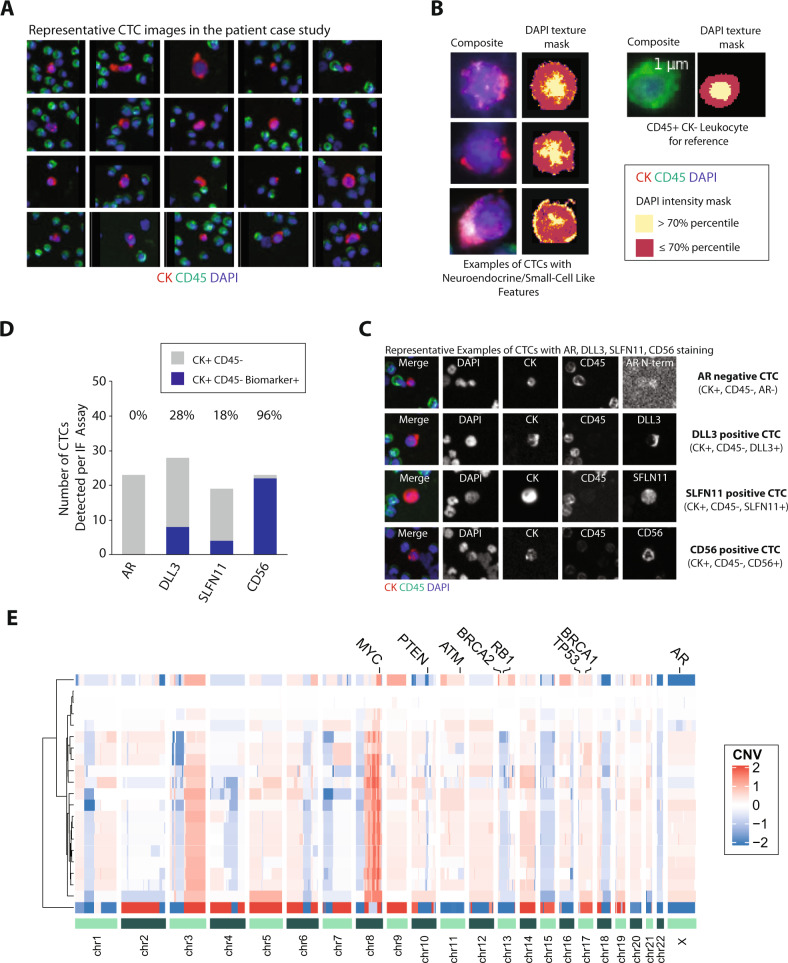
Fig. 2.

