# Supplementary material for: Circulating tumor cell heterogeneity in neuroendocrine prostate cancer by single cell copy number analysis
Source: NPJ Precis Oncol. 2021 Aug 12;5:76. doi: 10.1038/s41698-021-00211-1 (PMC8361159; doi:10.1038/s41698-021-00211-1)
Supplement: Supplementary file 16 — Supplementary Information [file 41698_2021_211_MOESM16_ESM.pdf]

Supplementary Table 1. CTC enumeration and DLL3 expression in Case Study and other cases included in the paper

				Epic Internal Accession ID	*CTC Enumeration*		*DLL3 Expression*	
					CK+ Cells, CK+ Clusters, CK- Cells, CK- Clusters, and Apoptotic Cells per milliliter of blood tested	*CK+ Cells and CK+ Clusters per milliliter of blood tested*	*DLL3+ CK+ Cells, CK+ Clusters, CK- Cells, CK- Clusters, and Apoptotic Cells per milliliter of blood tested*	*DLL3+ CK+ Cells and CK+ Clusters per milliliter of blood tested*
**Patient ID**	**Pathology**	**In Paper**	**SP347 Concentration (ug/mL)**	**Tube ID**	**All Populations/mL**	**Traditional CTCs/mL**	**DLL3+ All Populations/mL**	**DLL3+ Traditional CTC/mL**
10	CRPC-NE	Yes	1,0	3449	40,5	40,5	11	11
11	CRPC-Adeno	Yes	1,0	3699	70	70	0	0
12	CRPC-NE	Yes	1,0	5926	451,7	425,6	141,9	130,7
26	CRPC-NE	Yes	1,0	9266	121,2	116,9	118,6	114,3
31	CRPC-NE	Yes	1,0	30221	193,7	161,6	103,2	92,5
33	CRPC-Adeno	Yes	1,0	32769	13,5	10,6	0,6	0
35	CRPC-NE	Yes	1,0	33048	45,2	41,1	12,3	10,3
36	CRPC-NE	Yes	1,0	33698	45,8	41,6	4,2	4,2
37	CRPC-Adeno	Yes	1,0	34143	10,4	10,4	1,6	1,6
38	CRPC-NE	Yes	1,0	34142	29,5	26,2	4,4	3,3
39	CRPC-NE	Case Study	1,0	35172	16,1	14,2	3,8	3,8
41	CRPC-Adeno	Yes	1,0	38928	17,5	6,4	0,9	0

Abbreviations. CK, cytokeratins; CRPC-Adeno, prostate adenocarcinoma; CRPC-NE, castration-resistant prostate cancer with neuroendocrine differentiation; CTCs, circulating tumor cells; DLL3, Delta-like protein 3.
